# A Cascade Ensemble Learning Model for Human Activity Recognition with Smartphones

**DOI:** 10.3390/s19102307

**Published:** 2019-05-19

**Authors:** Shoujiang Xu, Qingfeng Tang, Linpeng Jin, Zhigeng Pan

**Affiliations:** 1 Virtual Reality and Intelligent Systems Research Institute, Hangzhou Normal University, Hangzhou 311121, China; shoujiang.xu@jsfpc.edu.cn (S.X.); tqf1013@stu.hznu.edu.cn (Q.T.); kinis1984@hznu.edu.cn (L.J.); 2 School of Information Engineering, Jiangsu Food and Pharmaceutical Science College, Huaian 223001, China

**Keywords:** human activity recognition, cascade ensemble learning model, smartphone, sensor, extremely gradient boosting trees, Random Forest, extremely randomized trees, Softmax Regression

## Abstract

Human activity recognition (HAR) has gained lots of attention in recent years due to its high demand in different domains. In this paper, a novel HAR system based on a cascade ensemble learning (CELearning) model is proposed. Each layer of the proposed model is comprised of Extremely Gradient Boosting Trees (XGBoost), Random Forest, Extremely Randomized Trees (ExtraTrees) and Softmax Regression, and the model goes deeper layer by layer. The initial input vectors sampled from smartphone accelerometer and gyroscope sensor are trained separately by four different classifiers in the first layer, and the probability vectors representing different classes to which each sample belongs are obtained. Both the initial input data and the probability vectors are concatenated together and considered as input to the next layer’s classifiers, and eventually the final prediction is obtained according to the classifiers of the last layer. This system achieved satisfying classification accuracy on two public datasets of HAR based on smartphone accelerometer and gyroscope sensor. The experimental results show that the proposed approach has gained better classification accuracy for HAR compared to existing state-of-the-art methods, and the training process of the model is simple and efficient.

## 1. Introduction

HAR refers to the process of computer detection, analysis and understanding of various human activities and behaviors through different machine learning algorithms. It has wide application prospects in the fields of virtual education and entertainment, sport injury detection, elderly care and rehabilitation, and smart home environment monitoring. Particularly, identifying daily activities is critical to healthy lifestyle maintenance and patient rehabilitation management, as well as helpful to detect and diagnose some specific diseases.

HAR technology usually utilizes different multi-modal data generated from various hardware devices to detect human posture, physical activity status and behavioral actions [1]. At present, the research on HAR mainly can be classified into several circumstances assisted by different technologies, such as video, wearable and mobile phone sensors, social networks, and wireless signals. Video-based methods primarily identify human activities by capturing images, video or camera surveillance [2]. As mobile phones and other wearable device sensors have evolved, inertial sensor data have been acquired using mobile or wearable embedded sensors placed on different body parts to infer details of human activity and postural transition. In addition, some people have recently suggested the social networking method [3], which is founded on the appropriate human profiles of multiple social network sources to understand a user’s behavior and interests. Recently, a new approach for HAR based on wireless signals [4] has been proposed.

However, due to the unique advantages of sensor data generated from smartphone or wearable devices, it plays an immeasurable role on human motion analysis, activity monitoring and detection. With the rapid advancement and popularization of smartphone technology, especially in the field of microelectronics and sensors, extracting knowledge from data acquired by ubiquitous sensors has become a very active research field [5]. Particularly, HAR using powerful sensors embedded in smartphones has received widespread attention in recent years, and the demand for applications in the research area of pervasive computing and mobile computing, surveillance-based security, environment-aware computing, and environment-assisted living has grown rapidly because of its efficient ability to recognize human activities [6]. A smartphone is almost a must-have item for everyone, usually equipped with various sensors such as accelerometer, gyroscope, thermometer, hygrometer, barometer, magnetometer, heart rate sensor, sound sensor, image sensor, and so on. Smartphone users access personal information through a variety of smartphone sensors to enhance the user experience. Motion sensors (accelerometer and gyroscope) provide important information to facilitate the identification and monitoring of user movement [7]. For example, according to the data of the accelerometer and the gyroscope, human activities (sitting, standing, walking, running, and lying) can be recognized efficiently.

This paper proposes an innovative machine learning approach for HAR. This method integrates XGBoost, Random Forest, ExtraTrees and Softmax Regression together, and a new hybrid CElearning model is constructed. Experiments showed that the proposed model can effectively identify the various human activities based on the data from the accelerometer and the gyroscope, and its validity and stability on the public dataset of HAR were verified.

## 2. Related Work

HAR with mobile phone and wearable device sensors is characterized by ubiquity, unobtrusiveness, low cost and ease of usability. Mobile phones have become a part of people’s daily life, possessing high penetration rate and portable characteristic. Recently, the application of inferring human activity based on sensor information of mobile phones and wearable devices is becoming more and more popular. Compared with video-based methods, mobile sensors use statistical and frequency-based features to identify details of human activity, effectively reducing computation time and computational complexity [8]. HAR with video sensors involves invading people’s privacy [9], and is also affected by changes in illumination, which can lead to performance degradation [10]. On the other hand, mobile and wearable sensor-based methods provide a better basis for real-time presentation of HAR systems. Moreover, mobile phones and wearable devices are location-independent, easy to deploy and cost-effective [11]. Considering the obvious advantages of mobile and wearable sensor based HAR, research on mobile and wearable sensor based HAR has become a hot research area [12]. There are many different sensor-based approaches for activity recognition, such as computational state space models [13], computational causal behavior models [14] and ontology-based hybrid approach [15,16]. Meanwhile, data-driven approaches have gained lots of attention recently.

The conventional data-driven HAR method with sensors usually designs the features manually, and then utilizes the data mining technology such as decision tree and multilayer perception to perform activity recognition [17,18]. However, such classifiers are not able to effectively distinguish very similar activities, such as going upstairs and going downstairs. Abidine [19] proposed a new approach for improving daily activity recognition combined with principal component analysis, linear discriminant analysis and improved weighted Support Vector Machine (SVM) to overcome the problems of non-informative sequence features and class imbalance. In [20], fusion of various sensors for HAR is explored, and the significance of the accelerometer and gyroscope is effectively analyzed. In [21], accelerometer and gyroscope data about 13 activities are collected, and machine learning algorithms such as multilayer perception, naive Bayes, logistic regression, K-nearest neighbor are used to infer the human activities based on handcrafted features extracted from the sensor data. Khan [22] suggested Wii-remote device for HAR, which is characterized by lightweight, small and easy to use properties. In [23], a public human activity dataset based on accelerometer and gyroscope is released, and 561 handcrafted features for every physical activity are extracted and used for HAR based on multiclass SVM. Recently, studies indicate that deep learning methods for HAR are superior to conventional HAR method with handcrafted features. For instance, Hassan [24] proposed a novel activity recognition method based on smartphone inertial sensors. Efficient handcrafted features including mean, median, autoregressive coefficients, etc. are first extracted from raw data. The features are further processed by a kernel principal component analysis and linear discriminant analysis, and then Deep Belief Network (DBN) based HAR is carried out with satisfying results obtained. All the above work for HAR is founded on the handcrafted features. At present, there is no general method for extracting and selecting manual features in different circumstances. In addition, it is difficult to identify similar activities due to the particularity of the experimental conditions.

Deep learning is a machine learning technique that can automatically discover the characteristics of raw data based on representation learning. In recent years, major breakthroughs have been made in image recognition [25], speech recognition [26], natural language processing [27] and medical health [28]. In recent years, different deep learning methods have been used for HAR based on mobile and wearable sensors; for instance, restricted Boltzmann machine, autoencoder, Convolutional Neural Networks (CNN), and recurrent neural network. Radu [29] proposed an improved restricted Boltzmann machine to achieve integrated learning of multi-sensor data fusion, which can effectively identify the hidden features of human activity. Li [30] recognized human activities using deep Stacked Autoencoder (SAE) with an additional Softmax layer. The intimate correlation between smartphone sensor data and individual health is finally established. In [31], a deep SAE is adopted to extract high-level features and a unified framework with integrated feature extraction and classifier training is designed for HAR. In [32], a deep CNN is utilized to perform efficient and effective activity recognition based on accelerometer and gyroscope data, which exploits the inherent characteristics of activities with time-series signals completely. In [33], a multi-modal CNN with 2D kernels in both convolutional and pooling layers is proposed to explore local and spatial dependency on multi sensors. Chen [34] proposed a feature extraction method based on long short-term memory neural network to recognize the human activities with accelerometer data. These deep learning methods can flexibly generate different deep learning models to implement automatic features learning. Deep learning methods for HAR based on mobile and wearable sensors have the characteristics of high accuracy performance, high flexibility and robustness, and overcome the shortcomings of traditional handcrafted feature extraction.

Deep forest [35], which is a performance-efficient decision tree ensemble learning method, was proposed as an alternative deep learning model in 2017. It shows high-performance advantages in handwriting recognition, music classification, image processing and some other classification problems. The method has the characteristics of easy training, less adjustable hyper-parameters, strong parallel computing ability and good performance even on small-scale training sets. The deep forest model is mainly composed of a cascade ensemble module and a multi-grained scanning module of which the basic classifier is Random Forest. However, it is well known that classifiers’ diversity is very crucial for ensemble module [36], which inspires this paper’s core idea of cascade ensemble learning with different classifiers. In this paper, combined with the cascade ensemble module of deep forest model, a deep CElearning model with multiple classifiers based on the accelerometer and gyroscope data of mobile wearable device is proposed, which can effectively identify human activities whether by handcrafted or automatic feature extraction. The proposed model with automatic feature extraction does not need to design manual features in advance and can be applied to HAR under different circumstances.

## 3. Overview of the Proposed HAR System

**Figure 1:**
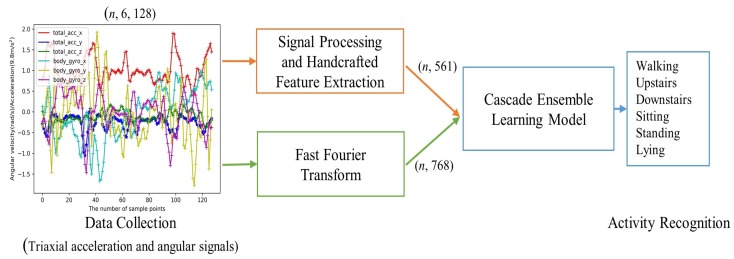
Overview of HAR system. Handcrafted feature extraction based HAR contains data collection, signal processing, feature extraction and CELearning model. Automatic feature extraction based HAR contains data collection, FFT and CELearning model.

The flowchart of the proposed HAR in this paper is shown in Figure 1. Sampled signals representing the different human activities are first obtained from the triaxial accelerometer and gyroscope sensors through data collection. The generated samples can be processed into a CELearning model for activity recognition in two different ways. The first way characterized by extracting 561 features manually is handcrafted feature extraction based HAR, as shown in the upper path of Figure 1. Meanwhile, the lower pipeline of the figure shows that the second one named by automatic feature extraction based HAR considers the frequency magnitudes of the sampled data through Fast Fourier Transform (FFT) as an input to the CELearning model for human activity classification.

### 3.1. Data Collection, Signal Processing and Handcrafted Feature Extraction

The data collection, signal processing and handcrafted feature extraction [23] are briefly discussed here. In this study, two prominent sensors, triaxial accelerometer and gyroscope, were separately used for data collection and the sampling rate of the raw signals was 50 Hz for both sensors. Median and low-pass Butterworth filters were used to reduce these signals’ noise. Then, the signals were sampled by a sliding window every 2.56 s, that is, there were precisely 128 sampling points corresponding to each axis of the two sensors within a window. Suppose the number of sliding windows is n, the data collected are depicted as (n, 6, 128).

Another low-pass Butterworth filter was applied to separate the acceleration signal into body acceleration and gravity information. Assuming that the gravitational force only has low frequency components, an optimal corner frequency 0.3 Hz was used to obtain gravity signal. More time signals were obtained from the triaxial signals by calculating the Euclidean and time derivatives, such as body acceleration jerk, body angular speed, body angular acceleration, body acceleration magnitude, gravity acceleration magnitude, body acceleration jerk magnitude, body angular speed magnitude, and body angular acceleration magnitude. Then, the corresponding frequency domain signals were generated through FFT and a total of 17 signals were obtained. Finally, more statistic operations were applied to the above time and frequency domain signals for manual feature extraction, and 561 features were calculated within a sampled window.

### 3.2. FFT in Lower Pipeline

FFT was used to further improve classification performance for activity recognition with three-axis acceleration and gyroscope sensors [30,32]. In this study, the denoised raw signals in the training set and testing set were transported to FFT module and the frequency domain magnitudes were considered as the input for classification because of its property of translation invariant. The process can be expressed as follows:

(1) $$\left\{\begin{array}{c} x_{tr}^{\prime }=\left|F_{6}\left(x_{tr}\right)\right|\\ x_{te}^{\prime }=\left|F_{6}\left(x_{te}\right)\right| \end{array}\right.$$

where *x*${}_{tr}$ and *x*${}_{te}$ are the signals sampled from the three-axis acceleration and the gyroscope sensors during the phase of data collection and depicted as the training and testing samples, respectively, and F_6 represents the FFT for the six different channels in signal *x*${}_{tr}$ and *x*${}_{te}$. | | is the element-wised modulus operation of each component of the vector, and the vectors *x*’${}_{tr}$ and *x*’${}_{te}$ are obtained. The FFT magnitudes has the same dimension with the original data and were considered as input to CELearning model for human activity classification.

## 4. CELearning Model

**Figure 2:**
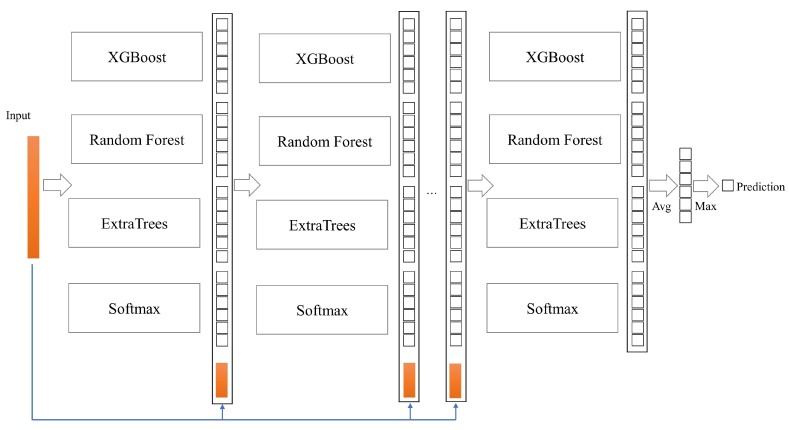
CELearning model. Each layer is composed of four basic classifiers which generate the probability vectors as augmented features for next layer’s learning.

In this paper, due to the particularity of HAR, a CELearning model composed of XGBoost, Random Forest, ExtraTrees and Softmax Regression is proposed to identify human activity features, as shown in Figure 2. In the CELearning model, the class vectors learned from the current layer concatenated with the original data are considered as input to the next layer, and the final prediction results are obtained through the last layer. In the process of training and testing, each of the four classifiers in each layer produces an estimate of probability distribution for the sample set. The estimated probability vector indicates the possibility of a sample’s belonging to different categories. For the six categories of HAR, in total, 24 augmented features are generated to facilitate the next layer’s classifiers’ learning. Each classifier’s performance in every layer is evaluated by k-fold cross validation, and the classification performance of each layer is obtained by fusion of four classifiers. If the classification performance does not improve obviously after several layers, the training process is terminated, and depth of the cascade layer is determined automatically.

### 4.1. XGBoost

XGBoost [37] is a supervised learning method that has been widely used in many fields. The XGBboost is composed of a set of Classification and Regression Trees. Assuming there exists *K* trees, the objective equations of the prediction and training models are Equations (2) and (3), respectively:

(2) $${\hat{y}}_{i}=\sum_{k=1}^{K}f_{k}\left(x_{i}\right)\;,\;\;\;\;\;\;\;\;\;\;\;\;\;f_{k}\in F$$

(3) $$Obj=\;\sum_{i=1}^{n}l(y_{i},{\hat{y}}_{i})+\sum_{k=1}^{K}\Omega (f_{k}),\;\;\;\;\;f_{k}\in F$$

In Equation (2), for a given *x*${}_{i}$, *f*${}_{k}$(*x*${}_{i}$) is the function value corresponding to each independent tree structure *k*, and ${\hat{y}}_{i}$ is the prediction value using *K* trees. The objective function of Equation (3) consists of training loss and regularization term. Since a class vector generated from the XGBoost in the proposed CELearning model represents an estimated class distribution, the loss function *l* here is defined as the Softmax loss function. The XGBoost uses an additive strategy to train the objective function. Assuming ${\hat{y}}_{i}^{\left(0\right)}=0$, a new decision tree is generated and the *t*-th output value ${\hat{y}}_{i}^{\left(t\right)}$ is obtained iteratively, as shown in Equation (4).

(4) $$\begin{array}{c} {\hat{y}}_{i}^{\left(0\right)}=0\\ {\hat{y}}_{i}^{\left(1\right)}=f_{1}\left(x_{i}\right)={\hat{y}}_{i}^{\left(0\right)}+\;f_{1}\left(x_{i}\right)\\ {\hat{y}}_{i}^{\left(2\right)}=f_{1}\left(x_{i}\right){+f}_{2}\left(x_{i}\right)={\hat{y}}_{i}^{\left(1\right)}+\;f_{2}\left(x_{i}\right)\\ \ldots \\ {\hat{y}}_{i}^{\left(t\right)}=\sum_{k=1}^{t}f_{k}\left(x_{i}\right)={\hat{y}}_{i}^{\left(t-1\right)}+f_{t}\left(x_{i}\right) \end{array}$$

Based on Equations (2) and (4), we take the Taylor expansion of the loss function up to the second order and the objective function becomes Equation (5).

(5) $${Obj}^{\left(t\right)}=\sum_{i=1}^{n}\left[l\left(y_{i},{\hat{y}}_{i}^{\left(t-1\right)}\right)+g_{i}f_{t}\left(x_{i}\right)+\frac{1}{2}h_{i}f_{t}^{2}\left(x_{i}\right)\right]+\Omega \left(f_{t}\right)+constant$$

where $g_{i}=\partial_{{\hat{y}}_{i}^{\left(t-1\right)}}l\left(y_{i},{\hat{y}}_{i}^{\left(t-1\right)}\right)$ and $h_{i}=\partial_{{\hat{y}}_{i}^{\left(t-1\right)}}^{2}l\left(y_{i},{\hat{y}}_{i}^{\left(t-1\right)}\right)$ are first- and second-order gradient statistics on the loss function.

We define the decision tree *f*${}_{t}$ as:

(6) $$f_{t}\left(x\right)=\omega_{q(x)},\;\omega \in R^{T},q:R^{d}\to \left\{1,2,\ldots ,T\right\}$$

where ω is the vector of scores on leaves of the decision trees, q(x) is a function assigning each data point to the corresponding leaf, and *T* is the number of leaves. Meanwhile, we define the regularization term representing the model complexity as:

(7) $$\Omega \left(f_{t}\right)=\frac{1}{2}\lambda \sum_{j=1}^{T}\omega_{j}^{2}+\gamma T$$

Define $I_{j}=\left\{i|q\left(x_{i}\right)=j\right\}$ as the set of indices of data points assigned to the *j*-th leaf. Based on Equations (5) and (7), the objective function becomes Equation (8), with constant term removed.

(8) $$\begin{array}{cc} {Obj}^{\left(t\right)} & =\sum_{i=1}^{n}\left[g_{i}f_{t}\left(x_{i}\right)+\frac{1}{2}h_{i}f_{t}^{2}\left(x_{i}\right)\right]+\gamma T+\frac{1}{2}\lambda \sum_{j=1}^{T}\omega_{j}^{2}\\ & =\sum_{j=1}^{T}\left[(\sum_{i\in I_{j}}g_{i})\omega_{j}+\frac{1}{2}\left(\sum_{i\in I_{j}}h_{i}+\lambda \right)\omega_{j}^{2}\right]+\gamma T \end{array}$$

For a fixed structure $q\left(x\right)$, the optimal $\omega_{j}^{*}$ of leaf *j* and the corresponding optimal value are calculated by Equations (9) and (10), respectively.

(9) $$\omega_{j}^{*}=-\frac{\sum_{i\in I_{j}}g_{i}}{\sum_{i\in I_{j}}h_{i}+\lambda }$$

(10) $${Obj}^{\left(t\right)}\left(q\right)=-\frac{1}{2}\sum_{j=1}^{T}\frac{{(\sum_{i\in I_{j}}g_{i})}^{2}}{\sum_{i\in I_{j}}h_{i}+\lambda }+\gamma T$$

Equation (10) can be used to measure the quality of a tree structure *q* as an evaluation and scoring function. Under normal circumstances, it is impossible to enumerate all possible tree structures *q*. In this method, a greedy algorithm is used to start from a single leaf and iteratively add the branches to the tree by finding the best split that makes the objective function smallest and the gain biggest. Suppose that *I*${}_{L}$ and *I*${}_{R}$ represent the instance sets of left and right nodes after the split, respectively, and $I=I_{L}+I_{R}$. Then, loss reduction after the split can be calculated by Equation (11) and can be used to select the best split from the split candidates.

(11) $${Obj}_{split}=\frac{1}{2}\left[\frac{{(\sum_{i\in I_{L}}g_{i})}^{2}}{\sum_{i\in I_{L}}h_{i}+\lambda }+\frac{{(\sum_{i\in I_{R}}g_{i})}^{2}}{\sum_{i\in I_{R}}h_{i}+\lambda }-\frac{{(\sum_{i\in I}g_{i})}^{2}}{\sum_{i\in I}h_{i}+\lambda }\right]-\gamma$$

### 4.2. Randomized Decision Trees

**Figure 3:**
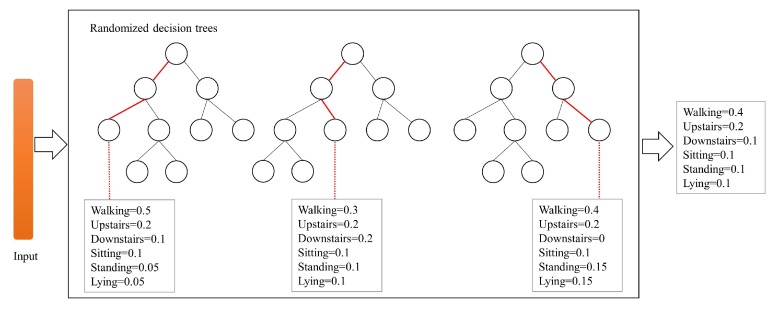
Class vector generation of the randomized decision trees. A probability vector is obtained from each decision tree and the final probability vector of randomized decision trees is jointly generated by all the decision trees.

The CELearning model proposed in this paper contains Random Forest [38] and ExtraTrees [39], which both belong to randomized decision trees and the schematic diagram of class vector generation of the two classifiers is shown in Figure 3.

For the Random Forest classifier, each Random Forest consists of several decision trees, and the features with the best *Gini* value are selected from candidate features for split. Each random decision tree produces an estimate of class distribution for a given sample, and the estimated class vectors of all decision trees in the Random Forest are averaged to obtain a final estimate of probability distribution as the class predication of Random Forest. In Figure 3, for six categories of HAR, the paths along which a sample instance traverses to leaf nodes are indicated by red and an estimated class distribution of the Random Forest is generated by all decision trees. ExtraTrees is similar to Random Forest and also composed of multiple decision trees. However, their two main differences are: (1) ExtraTrees completely randomly selects the features for split from all features, but Random Forest from the candidate features. (2) ExtraTrees utilizes all training samples to generate every decision tree, while Random Forest uses bootstrap samples.

### 4.3. Softmax Regression

The Softmax Regression model [40] is also integrated into the proposed CELearning model for HAR, and the class vector is considered as part of the augmented features. Softmax Regression is an extension of logistic regression, and can be easily used for multi-classification problems such as HAR. Given the sample *i*, the conditional probability of belonging to category *k* is:

(12) $$p\left(y^{i}=k|x^{i};\omega \right)=\frac{\exp({\left(\omega_{k}\right)}^{T}x^{i})}{\sum_{j=1}^{K}\exp({{(\omega }_{j})}^{T}x^{i})}$$

where $\omega_{k}$ is the weight of the sample *i* belonging to the category *k*. The purpose of the Softmax model is to estimate the parameter $\omega_{k}\left(1<=k<=n\right)$ based on training samples, and *n* is the number of features of the sample. Assuming that m training samples are independent of each other, then the parameters $\omega =\omega_{1},\omega_{2},\ldots ,\omega_{k}$ can be obtained by maximizing the following log-likelihood function:

(13) $$\begin{array}{cc} l\left(\omega \right) & =\sum_{i=1}^{m}\log\;p\left(y^{i}|x^{i},\omega \right)\\ & =\sum_{i=1}^{m}\sum_{k=1}^{K}I(y^{i}=k)\log\left(\frac{exp\left({\left(\omega_{k}\right)}^{T}x^{i}\right)}{\sum_{j=1}^{K}exp\left({{(\omega }_{j})}^{T}x^{i}\right)}\right) \end{array}$$

I(•) is an indicator function. I (Expression value is true) = 1, otherwise is 0. According to the gradient descent method, the optimal parameter ω can be calculated iteratively based on Equations (14) and (15):

(14) $$\omega_{j}=\;\omega_{j}+\alpha \nabla_{\omega_{j}}l\left(\omega \right),\;\;\;\forall j=1,2,\cdots K$$

(15) $$\nabla_{\omega_{j}}l\left(\omega \right)=\;-\frac{1}{m}\sum_{i=1}^{m}\left[x^{i}\left(I\left(y^{i}=j\right)-p\left(y^{i}=j|x^{i};\omega \right)\right)\right]+\lambda \omega_{j}$$

### 4.4. Augmented Features Generation

**Figure 4:**
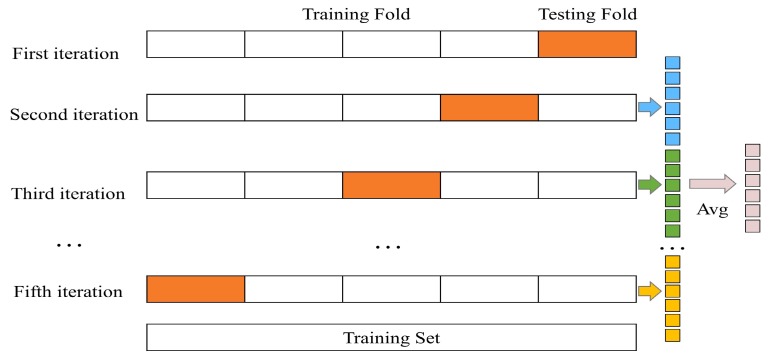
Augmented features generation of each classifier. K-fold cross validation is used for each classifier to generate K-1 estimated class vectors, which are averaged to obtain a final vector as augmented features.

To avoid over fitting, K-fold cross validation technique is adopted to generate the class vector for each classifier in a cascade layer. Every sample is used as training for K-1 times in the cross validation process and K-1 estimated class vectors, which are averaged to produce a final class vector as augmented features, are generated. As shown in Figure 4, for five-fold cross validation, a final class vector for the sample that belongs to test fold in the first iteration is calculated based on four estimated class vectors. Meanwhile, the final averaged class vector of each sample is considered as augmented features and concatenated with original data into next cascade layer for learning. For four classifiers in a cascade layer, four corresponding augmented vectors for a sample are generated in the process of K-fold cross validation and the classification accuracy prediction of current layer is calculated according to the basic idea of the final layer shown in Figure 2. If there is no obvious performance improvement about the classification accuracy prediction, the training process terminates, and the number of the cascade layer is automatically determined.

### 4.5. Confusion Matrix Definition

To facilitate the demonstration of the experimental results of HAR in this paper, a confusion matrix is defined as follows:

(16) $$F=\left[f_{ij}\right],F\in R^{\left(N_{C}+1\right)\times \left(N_{C}+1\right)},i,j\in 1,2,\cdots N_{C},N_{C}+1$$

(17) $$f\left(n\right)=\left\{\begin{array}{c} N_{ij},\frac{N_{ij}}{P};i,j\in \left\{1,2,\cdots ,N_{C}\right\}\\ \frac{N_{ii}}{\sum_{j=1}^{N_{C}}N_{ij}},1-\frac{N_{ii}}{\sum_{j=1}^{N_{C}}N_{ij}};i\neq N_{C}+1,j=N_{C}+1\\ \frac{N_{jj}}{\sum_{i=1}^{N_{C}}N_{ij}},1-\frac{N_{jj}}{\sum_{i=1}^{N_{C}}N_{ij}};i=N_{C}+1,j{\neq N}_{C}+1\\ \frac{\sum_{i=1}^{N_{C}}N_{ii}}{P},1-\frac{\sum_{i=1}^{N_{C}}N_{ii}}{P};i=N_{C}+1,j{=N}_{C}+1 \end{array}\right.$$

where *F* represents the confusion matrix and $f_{ij}$ is the unit value of the *i*th row and the *j*th column. Two values are included in a $f_{ij}$ unit: the total number and percentage of the specific samples, or the accuracy and inaccuracy rate. $N_{C}$ is the total number of categories, and equal to the number of activities. $N_{ij}$ is the total number of samples which belong to the *j*th class are predicted as the *i*th class. *P* represents the total number of the testing samples.

## 5. Experimental Results

**Figure 5:**
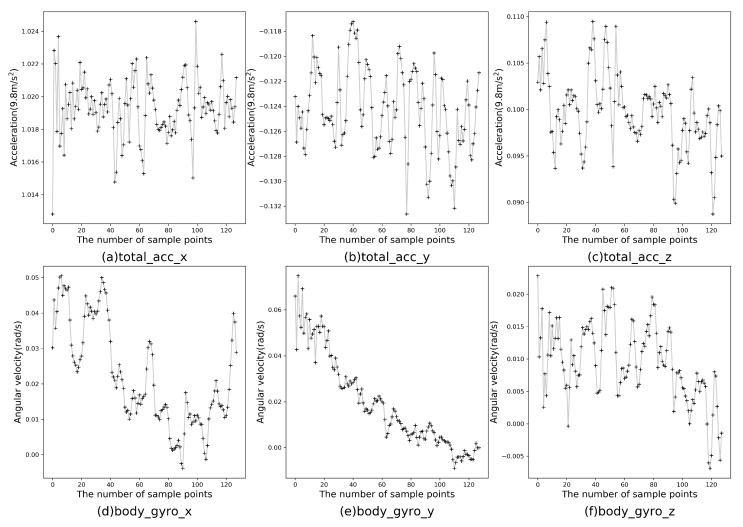
Three-axial linear acceleration and three-axial angular velocity: (**a**–**c**) the three-axis data of the accelerometer, respectively; and (**d**–**f**) the three-axis data of the gyroscope, respectively.

The experimental hardware configuration was as follows: CPU was Inter(R) Core (TM) i7-6700 3.40 GHz, and RAM was 8 GB. The operating system was Windows 10 and the programming language was Python 3.5. At present, there are many public wearable sensor-based datasets for HAR, such as HAR dataset [23], WISDM dataset [41], OPPORTUNITY dataset [42] and Cooking task dataset [43]. This study mainly conducted related experiments on the HAR dataset, which was completed by 30 volunteers aged 19–48 assisted by smartphone accelerometer and gyroscope, including six human activities (walking, going upstairs, going downstairs, sitting, standing and lying), and its extension dataset. The dataset was initially obtained from accelerometer and gyroscope on a smartphone fixed to the tester’s waist, including three-axis linear acceleration and three-axis angular velocity captured at a constant rate of 50 Hz. The HAR dataset was divided into 70% training data and 30% testing data, totalling 7352 and 2947, respectively. The persons in the training and testing sets were mutually exclusive, which means that the CELearning model did not know about the test persons to some extent in advance. The data size of one axis of the three-axis accelerometer and the three-axis gyroscope was 128, thus the length of one sample was 768. The first sample in the training set is shown in Figure 5, which consists of three-axial linear acceleration and three-axial angular velocity for 2.56 s and 128 sampling points.

The purpose of the experiment was to perform HAR using the CELearning model proposed in this paper, and to analyze and evaluate its classification performance. The proposed CELearning method in this paper includes four basic classifiers: XGBoost, Random Forest, ExtraTrees and Softmax Regression. The training set and testing set in the HAR dataset were used for training and testing in the proposed CELearning model, respectively. During the training process, the maximum number of the cascade layer was set in advance. At the same time, if the classification accuracy was not improved during recent three layers, the training process was terminated automatically, and the trained model was saved to facilitate the testing. To achieve better performance, the model with the best testing classification accuracy was generally obtained from several different experiments. To facilitate the comparison and present the advantages of the proposed model, the HAR experiments were carried out based on both handcrafted feature extraction and automatic feature extraction over HAR dataset.

### 5.1. HAR Based on Handcrafted Feature Extraction

Signal processing and feature extraction for each sample were performed, and 561 features were designed manually. The main hyper-parameters of the CELearning for HAR based on handcrafted feature extraction were set as follows: the number of trees of XGBoost was 30, maximum depth was 3, learning step size was 0.1 and the number of decision trees of the two randomized decision trees was 500. The remaining hyper-parameters were the default values in Scikit-learning.

After 10 consecutive training runs, the average accuracy of classification was 96.67%, and the performance was stable. Among them, the highest categorizing result of the HAR dataset is exhibited in Table 1, which represents a confusion matrix chart for the target class (true class) and predicted class (output class). In Table 1, the rows denote the predicted class, and the columns represent the true class. It was found that most activities could be effectively identified, even similar activities such as sitting and standing, upstairs and downstairs.

Compared with the multi-class SVM, Artificial Neural Network (ANN), DBN and SAE, the proposed model had higher classification accuracy. The classification accuracy rate was 0.38% higher than state of the art, as shown in Table 2.

### 5.2. HAR Based on Automatic Feature Extraction

In this experiment, to improve the classification performance, FFT was applied to generate magnitude vectors for each training and testing sample. For instance, 768 features consisting of six-dimensional (total_acc_x, total_acc_y, total_acc_z, body_gyro_x, body_gyro_y, body_gyro_z) sensor data were transformed into 768 corresponding new features with the same dimension (total_acc_x_fft, total_acc_y_fft, total_acc_z_fft, body_gyro_x_fft, body_gyro_y_fft, body_gyro_z_fft), which were considered as input.

The main hyper-parameters of the CELearning model based on automatic feature extraction were set as follows: the number of trees of XGBoost was 200, maximum depth was 5, learning step size was 0.1 and the number of decision trees of the two randomized decision trees was 500. The results of HAR classification are shown in Table 3.

In this experiment, the recognition accuracy between the similar activities was comparatively lower, such as walking upstairs and walking downstairs, sitting and standing. However, the overall accuracy of HAR was 95.93%, which was better than DBN, SAE and CNN, as shown in Table 4.

### 5.3. Convergence Analysis

**Figure 6:**
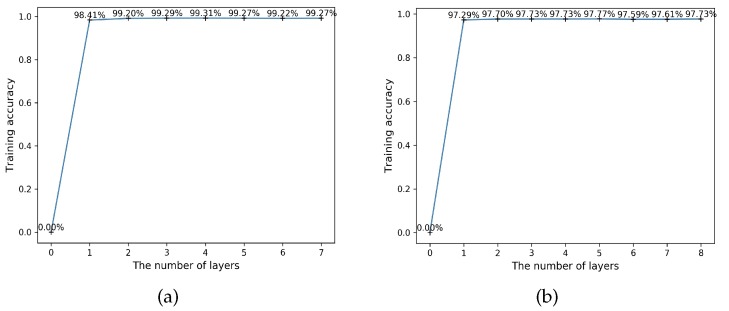
Convergence curves of the proposed model for HAR: (**a**) the convergence curve of handcrafted feature extraction based HAR; and (**b**) the convergence curve of automatic feature extraction based HAR.

The CELearning model proposed in this paper could converge timely and effectively during the training period. Figure 6 indicates the convergence curves of the two above methods for HAR. We found that much higher accuracy was obtained in the first iteration and the proposed CELearning model with different features extraction methods converged in the next few layers. For instance, the highest accuracy obtained (Figure 6a) was 99.31% and the model terminated when there was no further improvement in the following three iterations. Similar to the automatic feature extraction based model, the highest value was 97.77% (Figure 6b), which means the number of CELearning layers was 5. During the testing time, the final classification result was computed in the fifth layer.

### 5.4. Comparison of Different Combinations of Four Classifiers

We chose the CELearning model with four classifiers to infer human activities because of its high performance. Single classifier and CELearning model with different combinations of classifiers for HAR were analyzed and discussed. Statistical comparisons of 10 consecutive experiments of different combinations with same hyper-parameters setting detailed in Section 5.1 and Section 5.2 are shown separately in Table 5 and Table 6. The proposed model had higher accuracy and robust stability. Particularly, CELearning (Softmax Regression + ExtraTrees) was the best combination, which approached the highest performance with two different classifiers and CELearning (Softmax Regression+ ExtraTrees+ Random Forest) was one of the superior combinations with three different classifiers when using handcrafted feature extraction based HAR. Meanwhile, CELearning (XGBoost + Softmax Regression) and CELearning (XGBoost + Softmax Regression + Random Forest) achieved, respectively, the best performances with two and three classifiers when using automatic feature extraction based HAR.

As shown in Table 5 and Table 6, the proposed CELearning model with four classifiers achieved the best mean accuracy compared to other different combinations. The time cost of CELearning model with four classifiers was relatively higher than the four basic classifiers, and average time costs of proposed CElearning model based on different feature extraction methods were 15 min and 32 min, respectively. The differences between them are the size of input data and the hyper-parameters of XGBoost classifier. The process of tuning hyper-parameters was mainly based on experience. In the first step, the parameters of different classifiers were adjusted separately to obtain relatively high classification performance on the HAR dataset. The second step was to fine-tune the parameters of the four classifiers in the CELearning model to get more higher performance considering time cost. For instance, XGBoost could achieve high recognition accuracy around 94% based on handcrafted feature extraction when the number of trees was 200 and maximum depth was 3. However, when fine-tuning the parameters of the CELearning model, we found the CELearning model could obtain similar accuracy and was much less time-consuming when the number of trees was 30.

### 5.5. Human Activities and Postural Transitions Recognition

To further prove the effectiveness of the proposed CELearning model, a HAR experiment on the UCI public dataset ”Smartphone-Based Recognition of Human Activities and Postural Transitions Data Set” [44], which is an extension of the above HAR dataset, was carried out based on the handcrafted feature extraction. The dataset contained 12 categories composed of six basic activities and six postural transitions, and had 7767 training samples and 3162 testing samples. The 561 features were manually extracted from the data of the accelerometer and gyroscope sensor. We conducted some extra HAR experiments to prove the proposed model’s validity and experimental results are shown in Table 7. Main parameters are described in Section 5.1. The algorithm obtained the overall accuracy rate 95.10%, and was obviously superior to ANN and multi-class SVM in the classification performance.

## 6. Discussion

This paper proposes a CELearning model fusing four kinds of machine learning methods: XGBoost, Random Forest, ExtraTrees and Softmax Regression. This method possesses the characteristics of satisfying classification performance and simple training. It can be easily extended to other classification problems. The CELearning method concatenates the augmented features learned from each layer with the original data for relearning. In the learning process, combined with cross validation, the characteristics and advantages of each classifier are effectively utilized to further improve the classification performance.

Experiment results show that the model proposed in this paper had higher accuracy on two public HAR datasets than deep learning methods. We can see that the accuracy was over 95%, whether using handcrafted feature extraction-based or automatic feature extraction-based recognition method. In addition to its effectiveness and efficiency, the CELearning structure also showed its stability on the HAR based on smartphone sensors, signifying an important impact on reality application of wearable sensors. We found that the proposed algorithm could achieve around 95% accuracy when multiple consecutive experiments were conducted even with different parameters. The two kinds of feature extraction based HAR have their own superior characteristics. Handcrafted feature extraction based HAR using CELearning model is less time-consuming and has higher accuracy performance. However, automatic feature extraction based HAR does not need to design features in advance, which is more applicable to different HAR circumstances.

Our method still has some limitation and some pertinent improvements are needed to further promote the recognition performance. For instance, the recognition rate between similar activities is comparatively lower, especially the sitting activity is apt to be categorized as standing activity. In the future, we plan to exploit prior knowledge of human activities to distinguish the similar activities and improve the HAR performance. We also plan to combine the multi-grained scanning method of the deep forest algorithm to enhance the features learning and test the classification performance of HAR on more public datasets based on the multi-grained scanning and the CELearning model. At the same time, multi-grained scanning and the innovative CELearning proposed in this paper will be extended to other classification domain to verify the universality and effectiveness of the model.

## 7. Conclusions

In this study, we developed a CELearning model for HAR using smartphones. The data features were efficiently and effectively extracted from the accelerometer and gyroscope data obtained by the mobile and wearable device, and the classification accuracy of HAR was almost perfect on different datasets. Whether based on handcrafted features extraction or automatic features extraction, the CELearning model achieved the best classification accuracy performance compared with SVM, CNN and SAE. More importantly, the model could generalize across different circumstances and suite different datasets well.

## Figures and Tables

**Table 1:** Confusion matrix of HAR based on handcrafted feature extraction.

		Target Class						
		Walking	Upstairs	Downstairs	Sitting	Standing	Lying	Precision
**Predicted** **Class**	Walking	492 16.69%	22 0.75%	4 0.14%	0 0.00%	0 0.00%	0 0.00%	94.98% 5.02%
	Upstairs	1 0.03%	448 15.20%	17 0.58%	0 0.00%	0 0.00%	0 0.00%	96.14% 3.86%
	Downstairs	3 0.10%	1 0.03%	399 13.54%	0 0.00%	0 0.00%	0 0.00%	99.01% 0.99%
	Sitting	0 0.00%	0 0.00%	0 0.00%	464 15.74%	17 0.58%	0 0.00%	96.47 3.53%
	Standing	0 0.00%	0 0.00%	0 0.00%	27 0.92%	515 17.48%	0 0.00%	95.02% 4.98%
	Lying	0 0.00%	0 0.00%	0 0.00%	0 0.00%	0 0.00%	537 18.22%	100.00% 0.00%
	Recall	99.19% 0.81%	95.12% 4.88%	95.00% 5.00%	94.50% 5.50%	96.80% 3.20%	100.00% 0.00%	96.88% 3.12%

**Table 2:** Comparison of different methods based on handcrafted feature extraction.

Approach	Accuracy
ANN (as reported in [32])	91.08%
SVM [23]	96.00%
DBN (as reported in [30])	95.80%
SAE [30]	96.50%
CELearning (proposed)	96.88%

**Table 3:** Confusion matrix of HAR based on automatic feature extraction.

		Target Class						
		Walking	Upstairs	Downstairs	Sitting	Standing	Lying	Precision
**Predicted** **Class**	Walking	493 16.73%	3 0.10%	12 0.41%	0 0.00%	0 0.00%	0 0.00%	97.05% 2.95%
	Upstairs	1 0.03%	464 15.74%	27 0.92%	1 0.03%	0 0.00%	0 0.00%	94.12% 5.88%
	Downstairs	2 0.07%	4 0.14%	381 12.93%	0 0.00%	0 0.00%	0 0.00%	98.45% 1.55%
	Sitting	0 0.00%	0 0.00%	0 0.00%	432 14.66%	12 0.41%	0 0.00%	97.30% 2.70%
	Standing	0 0.00%	0 0.00%	0 0.00%	58 1.97%	520 17.65%	0 0.00%	89.97% 10.03%
	Lying	0 0.00%	0 0.00%	0 0.00%	0 0.00%	0 0.00%	537 18.22%	100.00% 0.00%
	Recall	99.40% 0.60%	98.51% 1.49%	90.71% 9.29%	87.98% 12.02%	97.74% 2.26%	100.00% 0.00%	95.93% 4.07%

**Table 4:** Comparison of different methods based on automatic feature extraction.

Approach	Accuracy
CNN [32]	95.75%
DBN (as reported in [30])	95.50%
SAE [30]	95.59%
CELearning (proposed)	95.93%

**Table 5:** Comparison of different combinations of four classifiers based on handcrafted feature extraction.

Approach	Mean Value (%)	Standard Deviation (%)
XGBoost	90.87	0.00
ExtraTrees	94.12	0.15
Random Forest	92.77	0.19
Softmax Regression	95.89	0.00
CELearning (Softmax Regression + ExtraTrees)	96.64	0.08
CELearning (Softmax Regression + ExtraTrees + Random Forest)	96.65	0.11
CELearning (proposed)	96.67	0.11

**Table 6:** Comparison of different combinations of four classifiers based on automatic feature extraction.

Approach	Mean Value (%)	Standard Deviation (%)
XGBoost	94.33	0.00
ExtraTrees	91.40	0.12
Random Forest	92.12	0.20
Softmax Regression	90.77	0.00
CELearning (XGBoost + Softmax Regression)	95.45	0.15
CELearning (XGBoost + Softmax Regression + Random Forest)	95.56	0.15
CELearning (proposed)	95.82	0.06

**Table 7:** Comparison of different methods for 12 categories of HAR.

Approach	Total Rightly Classified Samples	Overall Accuracy	Total Wrongly Classified Samples
ANN	2816	89.06%	346
SVM	2976	94.12%	186
CELearning	3007	95.10%	155

## Abbreviations

The following abbreviations are used in this manuscript:

ANN	Artificial Neural Network
CELearning	Cascade Ensemble Learning
CNN	Convolutional Neural Network
DBN	Deep Belief Network
ExtraTrees	Extremely Randomized Trees
FFT	Fast Fourier Transform
HAR	Human Activity Recognition
SAE	Stacked Autoencoder
SVM	Support Vector Machine
XGBoost	Extremely Gradient Boosting Trees
